# Selection and Validation of Reference Genes for Quantitative Real-Time PCR Normalization Under Ethanol Stress Conditions in *Oenococcus oeni* SD-2a

**DOI:** 10.3389/fmicb.2018.00892

**Published:** 2018-05-04

**Authors:** Shuai Peng, Longxiang Liu, Hongyu Zhao, Hua Wang, Hua Li

**Affiliations:** ^1^College of Enology, Northwest A & F University, Yangling, China; ^2^Shaanxi Engineering Research Center for Viti-Viniculture, Yangling, China; ^3^Heyang Experimental and Demonstrational Stations for Grape, Weinan, China

**Keywords:** *Oenococcus oeni*, reference gene, RT-qPCR, ethanol stress, normalization

## Abstract

The powerful Quantitative real-time PCR (RT-qPCR) was widely used to assess gene expression levels, which requires the optimal reference genes used for normalization. *Oenococcus oeni* (*O. oeni*), as the one of most important microorganisms in wine industry and the most resistant lactic acid bacteria (LAB) species to ethanol, has not been investigated regarding the selection of stable reference genes for RT-qPCR normalization under ethanol stress conditions. In this study, nine candidate reference genes (*proC, dnaG, rpoA, ldhD, ddlA, rrs, gyrA, gyrB*, and *dpoIII*) were analyzed to determine the most stable reference genes for RT-qPCR in *O. oeni* SD-2a under different ethanol stress conditions (8, 12, and 16% (v/v) ethanol). The transcript stabilities of these genes were evaluated using the algorithms geNorm, NormFinder, and BestKeeper. The results showed that *dnaG* and *dpoIII* were selected as the best reference genes across all experimental ethanol conditions. Considering single stress experimental modes, *dpoIII* and *dnaG* would be suitable to normalize expression level for 8% ethanol shock treatment, while the combination of *gyrA, gyrB*, and *rrs* would be suitable for 12% ethanol shock treatment. *proC* and *gyrB* revealed the most stable expression in 16% ethanol shock treatment. This study selected and validated for the first time the reference genes for RT-qPCR normalization in *O. oeni* SD-2a under ethanol stress conditions.

## Introduction

*Oenococcus oeni* (*O. oeni*) is well known as the main starter of malolactic fermentation (MLF). Through the MLF process, *O. oeni* reduces the acidity naturally and improves the quality and stability of wine (Guzzo et al., 2000; Maicas et al., 2000; Mohedano Mde et al., 2014). However, the harsh environmental conditions of wine such as high ethanol concentration, low pH, and SO_2_, can often delay the growth of *O. oeni* and MLF process (G-Alegría et al., 2004; Olguín et al., 2015; Betteridge et al., 2017). Ethanol stress is generally considered as one of the main inhibitors of *O. oeni* growth in wine (Mendoza et al., 2017; Contreras et al., 2018). The strain *O. oeni* SD-2a is able to survive and grow in high ethanol conditions and shows strong MLF ability (Liu, 2002), however its ethanol stress response mechanism was still obscure.

Quantitative real-time PCR (RT-qPCR), one of the most common technologies for quantifying gene expression level, was often used to analyze the stress response to abiotic and biotic stresses in *O. oeni* (Beltramo et al., 2006; Olguín et al., 2010). It is characterized by high sensitivity, specificity, good reproducibility and low cost, and always used to confirm results obtained by microarrays or RNA-seq (Allison et al., 2006; Fang and Cui, 2011). The methods of presenting quantitative gene expression include the absolute quantification method and relative quantification method. When using relative quantification in RT-qPCR, the optimal reference genes are indispensable in order to normalize RT-qPCR data of target gene. The MIQE guidelines (Bustin et al., 2009) emphasized the fact that the discovery of one or multiple stably expressed reference genes were crucial for obtaining accurate gene expression data. Therefore, the selection and validation of reliable reference genes for each particular condition are essential to quantitative accuracy.

Several stress-related genes such as *hsp18, citE, ctsR, clpP, ftsH*, have been studied using RT-qPCR analysis in *O. oeni* under different stress conditions, however only one common reference gene, *ldhD*, was used for normalization (Bourdineaud et al., 2003; Beltramo et al., 2006; Olguín et al., 2009, 2010; Capozzi et al., 2010). Recently, some studies attempted to elucidate the mechanisms of the adaptation and tolerance of *O. oeni* under stress conditions through transcriptomic and proteomic analysis, in which RT-qPCR was applied to confirm transcriptomic analysis results using the multiple common reference genes for normalization (Olguín et al., 2015; Margalef-Català et al., 2016; Liu et al., 2017). However, in these studies, the evaluation of reference genes was not performed in advance. As so far, few studies for evaluation of reference genes have been reported in *O. oeni* (Supplementary Table 1). However, the selection and validation of reference genes for RT-qPCR normalization in *O. oeni* under ethanol conditions have not yet been reported.

In this study, nine genes, *proC, rrs, dnaG, gyrA, ddlA, rpoA, gyrB, ldhD*, and *dpoIII*, were selected as candidate reference genes. Besides the commonly used genes for transcript normalization in *O. oeni* such as *ldhD, ddlA*, and *gyrB*, we selected others genes based on literature review. The three most diffused algorithms (geNorm, NormFinder, and BestKeeper) were used to calculate the expression stability of the candidate genes and obtain the most stable reference genes. In addition, the optimal reference genes were tested to normalize the expression of one target gene (*hsp18*) under ethanol stress conditions. This work is hoping to provide a basis for future study on gene expression under ethanol conditions in *O. oeni* SD-2a and other lactic acid bacteria (LAB) species.

## Materials and methods

### Bacterial strain

*Oenococcus oeni* SD-2a was obtained from our own collection (College of Enology, Northwest A&F University, Yangling, China). This strain was previously isolated from Shandong province in China and was properly identified (Liu, 2002; Li et al., 2006). The strain *O. oeni* SD-2a has obtained patent protection (02123444.2) in China.

### Growth conditions

The *O. oeni* SD-2a was cultured at 28°C and pH 4.8 in three flasks with 100 mL FMATB broth, which contains glucose 5 g/L, D, L-malate 5 g/L, yeast extract 5 g/L, peptone 10 g/L, MgSO4 •7H_2_O 0.2 g/L, MnSO4 •4H_2_O 0.05 g/L, Cysteine/HCl 0.5 g/L, and tomato juice 250 mL (Li et al., 2009; Liu et al., 2017). The growth of cultures was monitored by measuring OD value using a spectrophotometer (Cary 60 UV-Vis, Agilent Technologies, USA). When cultures reached the mid-exponential phase (OD600nm≈1, 10^9^ CFU/mL), they were completely mixed and split into three sterile flasks. Then the flasks were performed the shock treatment (ST) by adding with 8, 12 and 16% (v/v) ethanol, respectively. Bacterial samples were collected at time zero just before the addition of ethanol and then at one, 3 h after ethanol was added (Olguín et al., 2015). All assays above were performed in triplicate using independent cultures and incubated at 28°C and pH 4.8.

### RNA extraction and cDNA synthesis

Cells were harvested by centrifugation at 10,000 × g for 5 min at 4°C, the supernatants were removed and the pellets were washed with 10 mM Tris-HCl prepared with diethylpyrocarbonate-treated water (DEPC), which were then frozen in liquid nitrogen and kept at −80°C until RNA extraction (Margalef-Català et al., 2016). Total RNA extractions were performed using the E.Z.N.A.™ Bacterial RNA Kit (Omega Bio-tek, USA) that includes a DNase treatment step. The quality of the RNA samples was checked on a 1% (w/v) agarose gel (HydraGene, USA). RNA purity was characterized with optical density (OD) 260/280 and 260/230 ratios. All samples passed quality control with the ratio OD260/280 between 1.9 and 2.2 and ratio OD260/230<2.0. RNA concentration was determined by BioDrop μLite Spectrophotometer (Tamar Laboratory Supplies Ltd., Cambridge, England).

The cDNA was synthesized using the RevertAid First Strand cDNA Synthesis kit (Thermo Scientific) as described by the manufacturer. At the end of the reaction, each sample was diluted 1:100 with nuclease-free water prior to the RT-qPCR analysis.

### Candidate reference genes primers design

Primers of candidate reference genes and one target gene for RT-qPCR were designed using Primer Premier (version 5.0) and each has a length of about 20–25 bases, a G/C content of over 50% and a Tm of about 60°C (Beltramo et al., 2006). The primers were checked by gene-specific binding using the genome of *O. oeni* SD-2a (results not shown), which was also used to check exon-intron borders by matching the primers to their location. The primer sequences are listed in Table 1.

**Table 1:** Gene descriptions and primer sequences used for RT-qPCR.

**Gene Symbol**	**Annotation**	**LinRegPCR Efficiency**	**Sequence (5′-3′)**	**Tm(°C)**	**References**
*ldhD*	D-Lactate dehydrogenase	1.845	F-GCCGCAGTAAAGAACTTGATG	58.3	Margalef-Català et al., 2016
			R-TGCCGACAACACCAACTGTTT		
*dnaG*	DNA primase	2.041	F-TGTGGACGGAGTGGCAATGT	62	Desroche et al., 2005
			R-CGGTATTTTCTGTATATTTACTATCG		
*gyrA*	DNA Gyrase subunit A	1.930	F-CGCCCGACAAACCGCATAAA	62	Desroche et al., 2005
			R-CAAGGACTCATAGATTGCCGAA		
*gyrB*	DNA Gyrase subunit B	1.875	F- GGTTGAGGCTGGTCGAGTGTAT	62.3	This study
			R- GCATCCATCTCACCAAGTCCCT		
*rrs*	16S ribosomal RNA	1.847	F-ATGGTCGTCGTCAGCTCGTG	60.3	This study
			R- TGTGTTGCCCAGGTCATAAGG		
*ddlA*	D-Alanyl-alanine synthetase A	1.994	F- ATGGCAGTGGATGGTTTGACT	58.3	This study
			R-TAGTGTATTAGGCTCGCTTAGGAA		
*rpoA*	RNA polymerase subunit α	2.066	F- TGCTGGGAAGAAAGAAATGATG	56.3	This study
			R-AGTTAAACGAACGAACCGAAAG		
*proC*	Pyrroline-5-carboxylate reductase	1.876	F-CTGCTTGCTGATTGCGATTT	58	This study
			R-CCGTTAGTTCTTTAAGGCTTGTTG		
*dpoIII*	DNA polymerase III, alpha subunit	1.804	F-GCAGTGAAGGGACGCTTAAACG	62.3	Costantini et al., 2011
			R-ACCCAATCGCCTCGACATCATC		
*hsp18*	Heat shock protein Lo18	1.925	F-TGTGGACGGAGTGGCAATGT	60.3	Beltramo et al., 2006
			R-CGGTATTTTCTGTATATTTACTATCG		

### Quantitative real-time PCR

The RT-qPCR reactions were carried out on a Bio-Rad IQ5 Real-time PCR system with ChamQ SYBR qPCR Master Mix (Vazyme Biotech, Nanjing). Each reaction was performed in triplicate with a total reaction mixture of 20 μl final volume containing 2 μl diluted cDNA, 0.4 μl of each primer, 10 μl of ChamQ SYBR qPCR Master Mix, and 7.2 μl of RNase-free water. PCR conditions were indicated as follows: 95°C for 30 s, 40 cycles of 95°C for 10 s, 60°C for 30 s. To confirm product specificity, a melting curve analysis was performed after each amplification (Supplementary Figure 1). The threshold cycle (Ct) used in this study was automatically calculated by the Bio-Rad IQ5 Optical System software (version 2.1). The amplification efficiency was calculated from the raw data using LinRegPCR software (Ruijter et al., 2009; Tuomi et al., 2010).

### Statistical analysis

The algorithms geNorm (Vandesompele et al., 2002), NormFinder (Andersen et al., 2004), and BestKeeper (Pfaffl et al., 2004) were used to evaluate the expression stability of candidate reference genes under ethanol stress conditions. The raw Ct values were directly applied for BestKeeper analysis, however, for geNorm and NormFinder analysis, the raw data should be transformed into relative quantities using the 2^−ΔCt^ method: ΔCt = Ct sample − minimum Ct (Yan et al., 2014). Finally, an overall ranking of candidate reference genes was generated, by calculating the geometric mean of ranking orders from the three algorithms. In order to confirm the three major algorithms worked properly, the final overall ranking across all samples was compared to that obtained by a new software, named IdealRef (Palombella et al., 2017).

#### geNorm

geNorm(qbase+)is a statistical algorithm, which is based on the principle that the expression ratio of two reference genes should be constant in all samples, regardless of the experimental condition, or sampling time (Hellemans et al., 2007). The candidate reference genes were ranked by geNorm based on the expression stability value M, which is defined as the average pairwise variation with all other tested candidate genes. Lower *M*-values indicate more stable expression.

Using the geNorm algorithm, the normalization factor (NF) was also calculated by stepwise inclusion of a less stable gene until the (n+1)th gene has no significant contribution to the newly calculated normalization factor NF_n+1_(Vandesompele et al., 2002). Particularly, if the pairwise variation V_n/n+1_ between the two sequential normalization factors NF_n_ and NF_n+1_ is lower than the cut-off value of 0.15, it suggested that the NF_n+1_ is not required.

#### NormFinder

NormFinder is a Visual Basic application tool for Microsoft Excel used to calculate the stability values of each candidate reference genes by combining intra- and inter-group variations of gene expression (Andersen et al., 2004). The most stably expressed gene is the one with the lowest stability values and the lowest variation values of intra- and inter-groups.

#### BestKeeper

Pfaffl et al. (2004) have established Excel-based spreadsheet software named BestKeeper to determine the most stably expressed genes based on the standard deviation (*SD*) and the coefficient of variation (CV). The candidate reference genes can be ranked from the most stable one with the lowest variation, to the least stable one with the highest variation. Any candidate reference gene with the *SD* higher than 1 will be considered as inconsistent.

#### IdealRef

IdealRef is the algorithm recently developed by Palombella, based on the principle published by Vandesompele et al. (2002). The raw Ct values of candidate reference genes were required for this algorithm to calculate the average Ct and then the expression of one target gene was normalized with one reference gene at a time. From these values, the algorithm produces a dimensionless value (GS value), indicating the stability of the reference gene. The most stable gene is the one with the lowest GS value (Palombella et al., 2017).

### Validation of the selection of reference genes

In order to validate the selection of reference genes, the expression profile analysis of one target gene (*hsp18*) was carried out with the same cDNA samples used for the selection of reference genes (Guzzo et al., 2000; Beltramo et al., 2006). The RT-qPCR conditions were the same as previously described. The relative expression levels of the target gene were calculated according to the 2^−ΔΔCt^ method (Livak and Schmittgen, 2001). Samples collected at 0 h (without stress) were considered as control groups. Statistical significances between the two means were determined by the *t*-test using IBM SPSS Statistics version 22.0 (SPSS Inc., USA).

Expression was normalized using the three reference gene strategies in each treatment: (1) the optimal multiple reference genes from all samples, (2) the optimal multiple reference genes from each treatment, (3) the least stable reference gene from each treatment.

## Results

### Expression profiling of candidate reference genes

**Figure 1:**
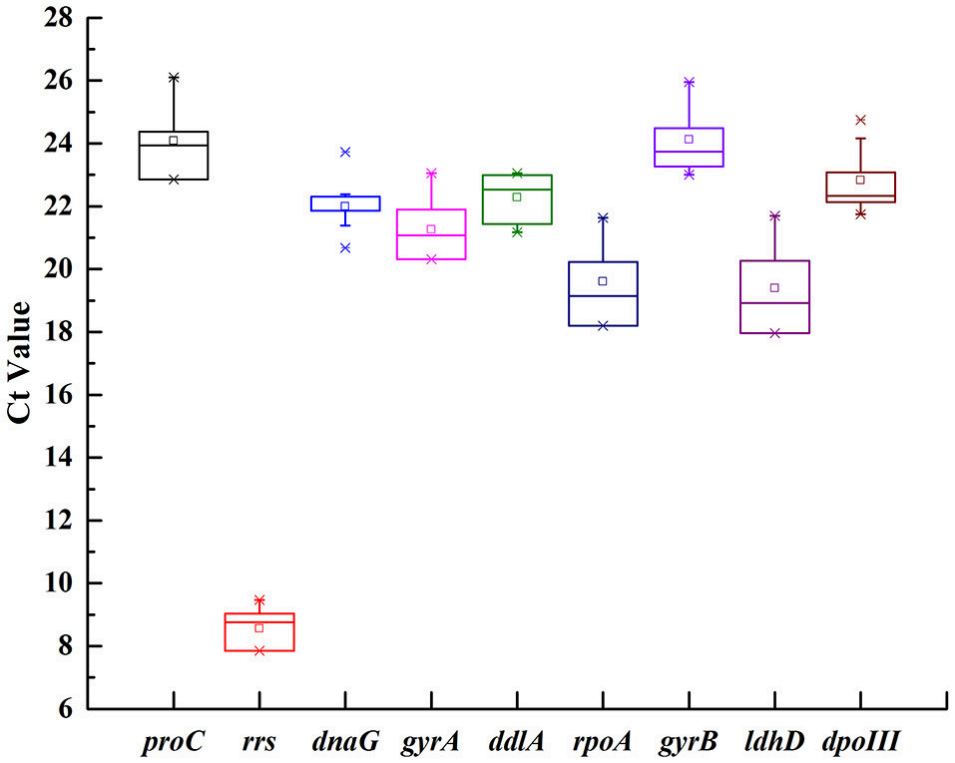
Expression levels of nine candidate reference genes across all samples in *O. oeni* SD-2a. The mean Ct values of nine candidate reference gene were described using a box and whiskers plot. The outer box is determined from 25 to 75th percentiles, and the inner box represents the mean value. The line across the box is the median. The whiskers represent percentiles from 5 to 95th, and outliers are depicted by asterisks.

In order to show transcriptional differences among nine candidate genes, the average Ct value was calculated across all experimental samples (Supplementary Table 2). The transcripts of these genes showed different levels of abundance (Figure 1). The mean Ct values for nine genes showed a range of variation from 8.57 to 24.12. *rrs* showed the most abundant transcript level with the lowest Ct value 8.57, while *proC* was the least abundant with the highest Ct value 24.12. *dnaG* revealed the least gene expression variation (coefficient of variation, CV, of 2.45%), while *rrs* and *ldhD* with CV of 6.61 and 6.34% were regarded as the most variable (Table 2).

**Table 2:** Gene expression stability ranked by geNorm, NormFinder, and BestKeeper in all samples and 8, 12, and 16% ethanol shock treatment.

**Group**	**Overall rank**	**Gene**	**geNorm**	***M*-value**	**Normfinder**	**Stability value**	**Bestkeeper**	***SD* (± Ct)**	**CV(% Ct)**
Total	1	*dnaG*	2	0.448	1	0.129	1	0.518	2.35
	2	*dopIII*	1	0.431	6	0.260	2	0.623	2.703
	3	*gyrA*	3	0.482	2	0.141	4	0.719	3.36
	4	*gyrB*	4	0.673	3	0.157	5	0.900	3.73
	5	*proC*	5	0.773	5	0.241	6	0.906	3.76
	6	*rrs*	7	0.879	4	0.166	7	0.543	6.34
	7	*ddlA*	9	0.999	9	0.547	3	0.699	3.14
	8	*ldhD*	6	0.83	8	0.510	9	1.196	6.13
	9	*rpoA*	8	0.917	7	0.365	8	1.151	5.87
8% ethanol ST	1	*dpoIII*	1	0.204	1	0.270	2	0.800	3.38
	2	*dnaG*	2	0.225	2	0.272	3	0.800	3.54
	3	*gyrA*	3	0.251	4	0.352	5	0.980	4.54
	4	*ddlA*	9	1.022	9	1.036	1	0.710	3.14
	5	*rrs*	7	0.802	3	0.349	4	0.310	3.78
	6	*gyrB*	6	0.735	5	0.497	6	1.140	4.56
	7	*rpoA*	4	0.863	6	0.527	8	1.180	5.92
	8	*ldhD*	5	0.573	8	0.863	9	1.210	6.04
	9	*proC*	8	0.632	7	0.747	7	1.390	5.57
12% ethanol ST	1	*gyrB*	4	0.503	3	0.296	1	0.270	1.15
	2	*gyrA*	3	0.411	1	0.130	4	0.560	2.65
	3	*rrs*	1	0.32	4	0.434	7	0.510	5.97
	4	*proC*	2	0.334	6	0.479	3	0.590	2.5
	5	*dopIII*	5	0.625	5	0.467	2	0.510	2.27
	6	*dnaG*	6	0.673	2	0.150	6	0.660	3.05
	7	*ddlA*	7	0.74	7	0.768	5	0.670	3.03
	8	*ldhD*	8	0.882	9	0.880	8	1.230	6.3
	9	*rpoA*	9	0.975	8	0.794	9	1.320	6.71
16% ethanol ST	1	*proC*	1	0.216	1	0.025	2	0.470	2
	2	*gyrB*	2	0.242	1	0.025	3	0.520	2.15
	3	*dnaG*	5	0.504	4	0.248	1	0.330	1.51
	4	*rrs*	3	0.279	3	0.081	9	0.620	7.09
	5	*gyrA*	4	0.391	5	0.250	5	0.620	2.88
	6	*dopIII*	7	0.631	7	0.615	6	0.700	3.03
	7	*ddlA*	9	0.881	9	1.038	4	0.610	2.77
	8	*rpoA*	8	0.727	6	0.575	7	0.700	3.68
	9	*ldhD*	6	0.572	8	0.907	8	0.760	4.02

*SD (± Ct): standard deviation of the Ct; CV (%Ct), coefficient of variance expressed as a percentage of the Ct level; ST, shock treatment*.

### Expression stability analyses

#### geNorm analysis

**Figure 2:**
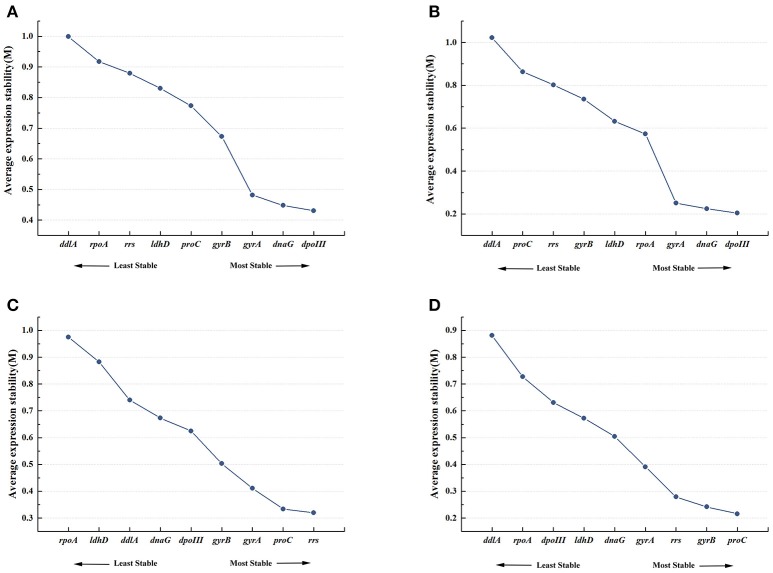
Expression stability and ranking of nine candidate reference genes by geNorm. Average expression stability (M) was calculated following stepwise exclusion of the least stable gene across all treatment groups. **(A)** All samples. **(B)** 8% ethanol shock treatment (ST). **(C)** 12% ethanol ST. **(D)** 16% ethanol ST.

Figure 2A showed the rank order of the candidate reference genes according to their expression stability (*M*-value) across all samples pooled together. When all 21 samples were analyzed together, *dpoIII, dnaG*, and *gyrA* were defined as the most stable genes with the *M*-value of 0.431, 0.448, and 0.482, respectively. While *rpoA* and *ddlA* were defined as the least stable genes with the *M*-value of 0.917 and 0.999, respectively. In 8% ethanol, we also found that *dpoIII, dnaG*, and *gyrA* (*M*-value, 0.204, 0.225, and 0.251, respectively) were the most stable genes, whereas *proC* and *ddlA* (0.863 and 1.022, respectively) were the least stable genes (Figure 2B). However, in 12% ethanol, the top-ranked genes were *rrs, proC*, and *gyrA* (*M*-value, 0.32, 0.334, and 0.411, respectively). *ldhD* and *rpoA* (*M*-value,0.882 and 0.975, respectively) showed the greatest variation, which were ranked eighth and ninth, respectively (Figure 2C). In 16% ethanol, *proC, gyrB*, and *rrs* (*M*-value, 0.216, 0.242, and 0.279, respectively) were identified as the most stable genes, while *rpoA* and *ddlA* (*M*-value, 0.727 and 0.876, respectively) as the least stable genes (Figure 2D). In addition, the *M*-values for all candidate reference genes were below the geNorm default limit of 1.5, indicating relatively high stability for all measured genes. The rank orders generated by geNorm analysis were shown in Table 2.

#### NormFinder analysis

NormFinder analysis result was slightly different from that generated by geNorm (Table 2). According to NormFinder analysis, *dnaG* and *gyrA* were ranked in top positions across all samples, but ranked second and third by geNorm, respectively. In 8% ethanol, *dpoIII, dnaG*, and *rrs* were ranked in top positions by NormFinder analysis, while *rrs* was ranked seventh by geNorm. In 12% ethanol, according to NormFinder, *gyrA, dnaG*, and *gyrB* were considered as the most stable genes, while *dnaG* was ranked sixth by geNorm. In 16% ethanol, *gyrB* and *proC* were ranked in top position by NormFinder, which was identical with the result by geNorm. Nevertheless, in all samples or each treatment, the least stable genes identified by both methods were consistent.

#### BestKeeper analysis

As shown in Table 2, across all samples, the most stably expressed gene identified by BestKeeper were *dnaG* and *dpoIII*, which were also ranked in top position by geNorm, however *dpoIII* was ranked sixth position by NormFinder. In 8% ethanol, *ddlA, dpoIII*, and *dnaG* were regarded as the most stably expressed genes by BestKeeper, while *ddlA* as the least stable by both geNorm and NormFinder. Furthermore, in 12% ethanol, we found that *gyrB* was the most stably expressed using BestKeeper analysis, while ranked fourth and third by geNorm and NormFinder, respectively. And the same lowest stably expressed genes, r*poA* and *ldhD*, were emerged by the three algorithms. In 16% ethanol, *dnaG* was emerged as the most stably expressed by BestKeeper, followed by *proC* and *gyrB*, however *dnaG* was ranked fifth and fourth by geNorm and NormFinder analysis, respectively. Whereas the least stable gene identified by BestKeeper was *rrs*, which was ranked third by both geNorm and NormFinder.

**Figure 3:**
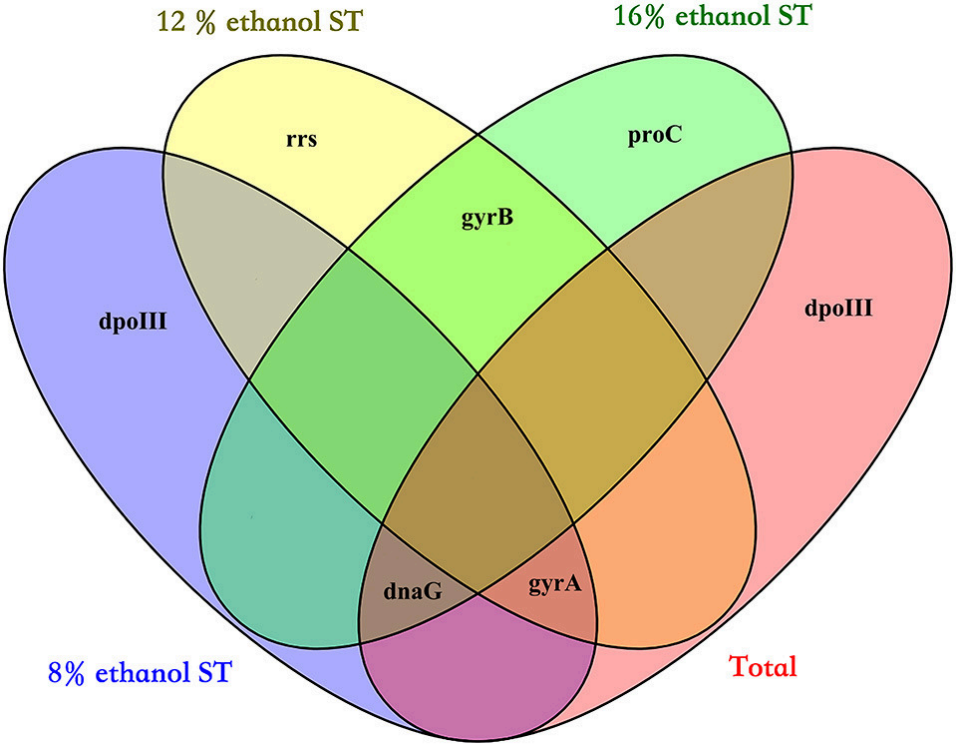
Venn diagram showing the overlap of the three most stable genes in all samples (Total), 8% ethanol shock treatment (ST), 12% ethanol ST, and 16% ethanol ST. The three most stable genes were selected by the overall ranking of three algorithms (geNorm, NormFinder, and BestKeeper).

Finally, by calculating the geometric mean of the rank orders generated by the three algorithms, the overall rankings in all samples and each treatment were obtained and shown in Table 2: the three most stable reference genes were respectively *dnaG, dpoIII*, and *gyrA* in all samples, *dpoIII, dnaG*, and *gyrA* in 8% ethanol, *gyrA, gyrB*, and *rrs* in 12% ethanol, and *proC, gyrB*, and *dnaG* in 16% ethanol. As shown in Figure 3, no universal reference was found for all experimental conditions in this study.

#### IdealRef analysis

In order to confirm the three major algorithms worked properly, the raw Ct data of all samples were analyzed with *hsp18* as target gene using IdealRef software. The most stable reference genes (*dpoIII* and *gyrA*) identified by IdealRef software were consistent with those selected by the overall ranking across all samples (Supplementary Table 3).

### Determination of the optimal number of reference genes for normalization by geNorm

**Figure 4:**
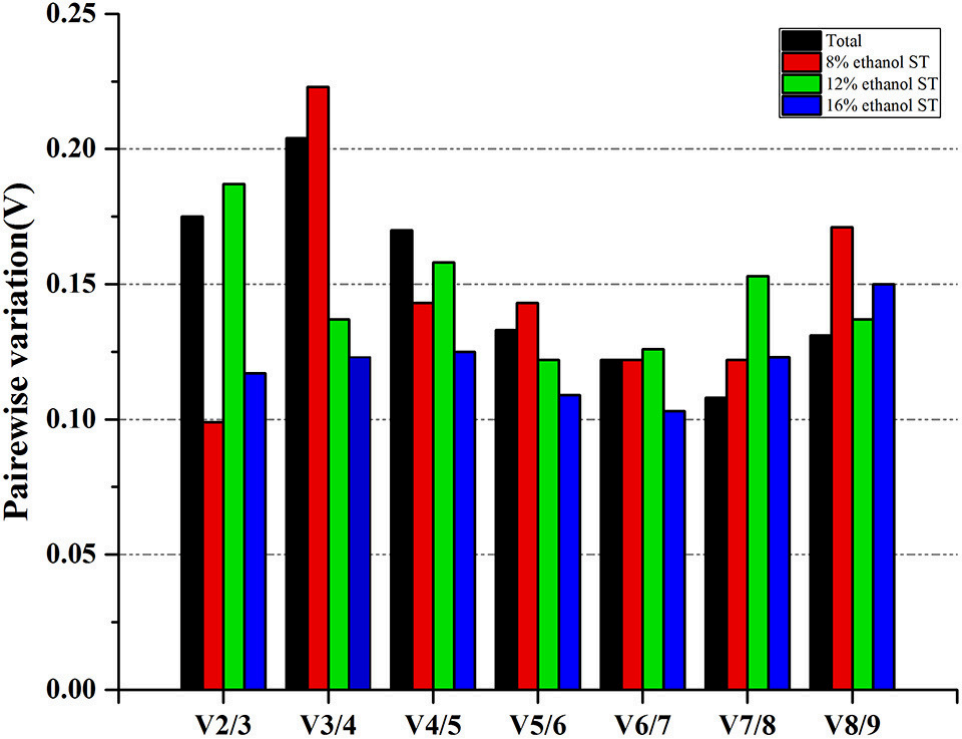
Determination of the optimal number of reference genes for normalization. Pairwise variation (V_n_/V_n+1_) analysis between the normalization factors (NF_n_ and NF_n+1_) was performed by geNorm to determine the optimal number of reference genes.

In order to determine the optimal number of reference gene used for accurate normalization, geNorm performed a stepwise calculation of the pairwise variation (V_n_/V_n+1_) between two sequential normalization factors (NF_n_ and NF_n+1_). As shown in Figure 4 and Supplementary Table 4, in 8 and 16% ethanol, the V_2/3_ values were respectively 0.099 and 0.117, lower than the cut-off value of 0.15, indicating that two genes would be sufficient for normalization under these conditions. Therefore, we could consider *dpoIII* and *dnaG* as the optimal multiple reference genes in 8% ethanol, while *proC* and *gyrB* in 16% ethanol. However, in 12% ethanol, three reference genes were required for normalization with an acceptable V_3/4_ value of 0.137. Hence, *gyrA, gyrB*, and *rrs* would be used as the optimal multiple reference genes for normalization in 12% ethanol.

### Validation of the selection of reference genes

An expression profile analysis of the target gene (*hsp18*) was carried out to validate the selection of candidate reference genes. According to the reference gene strategies mentioned above, besides the optimal multiple reference genes selected from each treatment, we also used the least stable gene in each treatment: *proC* in 8% ethanol, *rpoA* in 12% ethanol and *ldhD* in 16% ethanol, and the optimal multiple reference genes selected from all samples: *dpoIII* and *gyrA* in 8% ethanol, *dnaG, dpoIII*, and *gyrA* in 12% ethanol and *dnaG* and *dpoIII* in 16% ethanol, to normalizer the expression level of *hsp18*.

**Figure 5:**
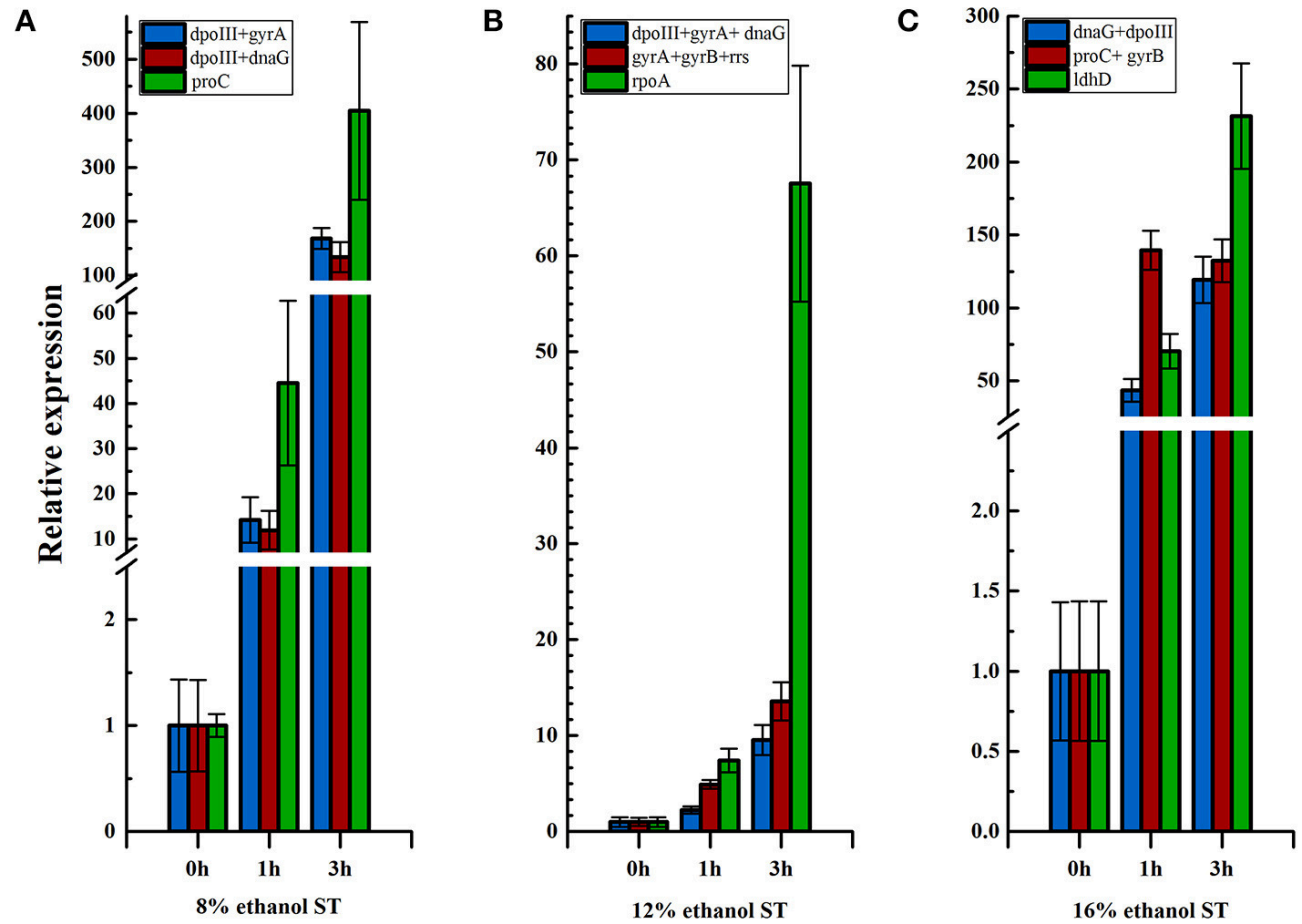
Relative expression of *hsp18* in different ethanol stress conditions. **(A)** Relative expression of *hsp18* in 8% ethanol shock treatment (ST) normalized using reference genes, *dpoIII*+*gyrA, dpoIII*+*dnaG*, and *ldhD*, respectively. **(B)** Relative expression of *hsp18* in 12% ethanol ST normalized using reference genes, *dpoIII*+*gyrA*+*dnaG, gyrA*+*gyrB*+*rrs*, and *rpoA*, respectively. **(C)** Relative expression of *hsp18* in 16% ethanol ST normalized using reference gene*, dnaG*+*dpoIII, proC*+*gyrB*, and *ldhD*, respectively. The results are represented as mean fold changes in relative expression compared to control samples (t = 0 h).

As shown in Figure 5 and Supplementary Table 5, the expression level of *hsp18* increased continuously at 1 and 3 h in 8% ethanol, using *dpoIII* and *dnaG* as the optimal reference genes. Similar expression pattern was also revealed in 12% ethanol when normalization was carried out using *dnaG, dpoIII*, and *gyrA* as the optimal reference genes. In contrast, when normalizing using *proC* as reference gene, which was the least stable reference gene in 8% ethanol, the expression exhibited also continuously but sharply increasing. Meanwhile the relative expression was obviously overestimated, much higher than when normalizing using the optimal reference genes, *dpoIII* and *dnaG*, at all time points. The same expression trend was observed in 12% ethanol when normalizing using the least stable reference gene, *rpoA*. Normalizations with the optimal reference genes selected from all samples were also respectively performed in 8 and 12% ethanol. The expression levels were consistent with those obtained when normalization with the optimal reference genes from each treatment. However, in 16% ethanol, when normalization was performed using *proC* and *gyrB* as the optimal reference genes, the relative expression increased sharply at 1 h and unchanged at 3 h. The relative expression showed a different trend compared to when normalized with the least stable gene, *ldhD*, or with the optimal reference genes from all samples, *dnaG* and *dpoIII*.

## Discussion

As the most resistant LAB species to ethanol, *O. oeni* has evolved different mechanisms to cope with the ethanol stress condition in wine (Guzzo et al., 2000; Beltramo et al., 2004; G-Alegría et al., 2004; Li et al., 2009; Olguín et al., 2009, 2015). However, the molecular mechanisms of ethanol tolerance were still not well understood. RT-qPCR is a powerful tool to reveal the stress tolerance mechanisms of *O. oeni*, but the accuracy of its result was directly affected by the expression stability of reference genes (Vandesompele et al., 2002; Bustin et al., 2009; Sumby et al., 2012). Thus, the selection of the optimal reference genes is essential for quantification of gene expression.

In our study, three popular statistical algorithms, geNorm, NormFinder, and BestKeeper, were used for the evaluation of the expression stability of nine candidate reference genes in three different ethanol conditions. geNorm confirmed the most stable gene with a low pairwise variation among reference genes (Vandesompele et al., 2002). NormFinder determined the most stable gene with the lowest stability value calculated by combining the intra- and inter-group variation (Andersen et al., 2004), and BestKeeper did this based on the standard deviation and the coefficient of variation (Pfaffl et al., 2004). The final rankings of candidate reference genes were not identical when using different algorithms in this study. The variance in results provided by different algorithms was also reported in the previous works (Velada et al., 2014; Gao et al., 2017). The main reason causing the discrepancy is the varying priorities in different algorithms. Furthermore, co-regulated genes with similar expression profiles can affect the rank order of geNorm, resulting in making the wrong choice for normalization. In contrast, the algorithms of NormFinder and Bestkeeper are less sensitive to co-regulation (Wu et al., 2016). Thus, we selected the most stable reference genes based on the integrating of the results from three algorithms. By combining the results of three algorithms, we found that *dnaG, dpoIII*, and *gyrA* were the most stable reference genes when all samples pooled were analyzed together, however different experimental conditions emerged their own optimal reference genes. This is more evident that the validation of the reference genes for specific experimental condition is required prior to use in RT-qPCR normalization. In 8% ethanol, *dpoIII* performed as the most stable gene, followed by *dnaG* and *gyrA*. In 12% ethanol, *gyrA* and *gyrB* were identified as the most stable gene, while *rrs* ranked third position. In 16% ethanol, the top-ranked gene was *proC*, which was not evaluated in the previous studies of *O. oeni*, whereas *gyrB* and *dnaG* were ranked second and third respectively. In our study, *dpoIII, dnaG, gyrB*, and *gyrA* showed better expression stability, while in some studies, they were used together as reference genes for RT-qPCR normalization in *O. oeni*, but not evaluated particularly (Costantini et al., 2011; Margalef-Català et al., 2016; Liu et al., 2017). Moreover, the traditional reference genes in *O. oeni*, such as *ddlA* and *ldhD* varied greatly in our experimental conditions. *ddlA* were ranked fourth, seventh and seventh in the overall rangkings of 8, 12, and 16% ethanol, respectively. *ldhD*, together with *rpoA*, was considered as the least stable genes in all experimental conditions, so in this work, it was not chosen as reference gene for the normalization. This result is consistent with that described by Cafaro et al. (2014). *rrs*, encoding 16S rRNA, has been reported unstable in some studies with the disadvantage of high transcript level (Desroche et al., 2005; Wen et al., 2016). Our results showed also the much higher abundance of *rrs* than other genes, however in 12% ethanol, the expression stability of *rrs* was stable, which was ranked in top position by geNorm analysis and third in the overall ranking, was good (Figure 2C). Thus, in this study, *rrs* would be used for normalization to validate the selection result in 12% ethanol. Furthermore, in order to neutralize the impact of the high abundance of *rrs*, the templates for *rrs* should be diluted more times than the samples for the other genes in RT-qPCR analysis, however the different dilution ratios can lead to more human errors (Sun et al., 2016). Thus, in each experiment, three independent biological replicates and three technical replicates were required at least.

In order to validate the selection of reference genes, in this study, we employed three reference gene strategies to normalize the relative expression of one stress response gene, *hsp18*. In this study, the results showed that the expression patterns were influenced by the reference gene strategy obviously. When being normalized with the least stable gene, the relative expression levels of *hsp18* were overestimated in all experimental conditions (Figure 5). The expression overestimated would deeply affect the accurateness of analysis. The *hsp18* gene acts in the early response to stress conditions (Beltramo et al., 2006). A significant over-expression of this gene was observed for 1 h after ethanol shock in all experimental conditions when normalization with the optimal reference genes from each treatment or from all samples. Furthermore, when normalization with the optimal reference genes, in 8 and 12% ethanol, the expression trends were consistent with those observed in previous studies (Guzzo et al., 2000; Beltramo et al., 2006). However, in 16% ethanol, the expression trend using *proC* and *gyrB* as reference genes was different from using *dnaG* and *dpoIII* (Figure 5C). These results further verified the importance of the selection of reliable reference genes for each particular condition. The *hsp18* gene was proposed as molecular marker to select good MLF starters by Coucheney et al. (2005). Capozzi et al. (2010) reported that strains with better MLF performance presented higher relative expression of *hsp18*, which confirmed *hsp18* as a useful tool to evaluate the ability of *O. oeni* strains to survive in wine and to perform MLF. Moreover, Betteridge et al. (2017) considered *hsp18* as an indicator to determine the high ethanol tolerance phenotype of *O. oeni*. Therefore, according to the expression results, *O. oeni* SD-2a seems to be a high ethanol tolerance strain and a good starter for MLF process.

Concluding, microarray and RNA-seq datasets from *O. oeni* could be used as alternative sources to identify novel candidate reference genes, however the validation of these novel genes using RT-qPCR or literature-based searches was required (Alexander et al., 2012; Pombo et al., 2017). The transcriptomic analysis of *O. oeni* SD-2a using RNA-seq has been reported by Liu et al. (2017). However, prior to validation of this transcriptomic analyses using RT-qPCR, the evaluation of reference genes for normalization was not performed. In further analysis, our findings will make the validation of high-throughput data from *O. oeni* SD-2a under ethanol stress condition more accurate and robust. In this study, we selected nine candidate genes, commonly used for normalization in *O. oeni*, based on literature review. These genes are still very popular with the researchers and still used for RT-qPCR data normalization in many studies.

This study is the first to systematically analyze reference genes for RT-qPCR under ethanol stress conditions in *O. oeni*. The results will benefit future gene expression studies in *O. oeni* SD-2a and facility the selection of reference genes of other LAB strains under ethanol condition.

## Author contributions

HL and SP conceived the idea of the study. SP, LL, and HZ designed and performed the experiments. SP and HW analyzed the data and wrote the paper. HL revised the paper.

### Conflict of interest statement

The authors declare that the research was conducted in the absence of any commercial or financial relationships that could be construed as a potential conflict of interest.
