# Supplementary material for: Selection and Validation of Reference Genes for Quantitative Real-Time PCR Normalization Under Ethanol Stress Conditions in Oenococcus oeni SD-2a
Source: Front Microbiol. 2018 May 4;9:892. doi: 10.3389/fmicb.2018.00892 (PMC5946679; doi:10.3389/fmicb.2018.00892)

Supplementary Material

Selection and validation of reference genes for quantitative real-time PCR normalization under ethanol stress conditions in *Oenococcus oeni* SD-2a

Shuai Peng, Longxiang Liu, Hongyu Zhao, Lin Yuan, Hua Wang*, Hua Li*

*** Correspondence:** Hua Li: lihuawine@nwafu.edu.cn Hua Wang: wanghua@nwafu.edu.cn

Supplementary Figure 1. Melting curves for nine candidate reference genes and one target genes, showing single peaks.

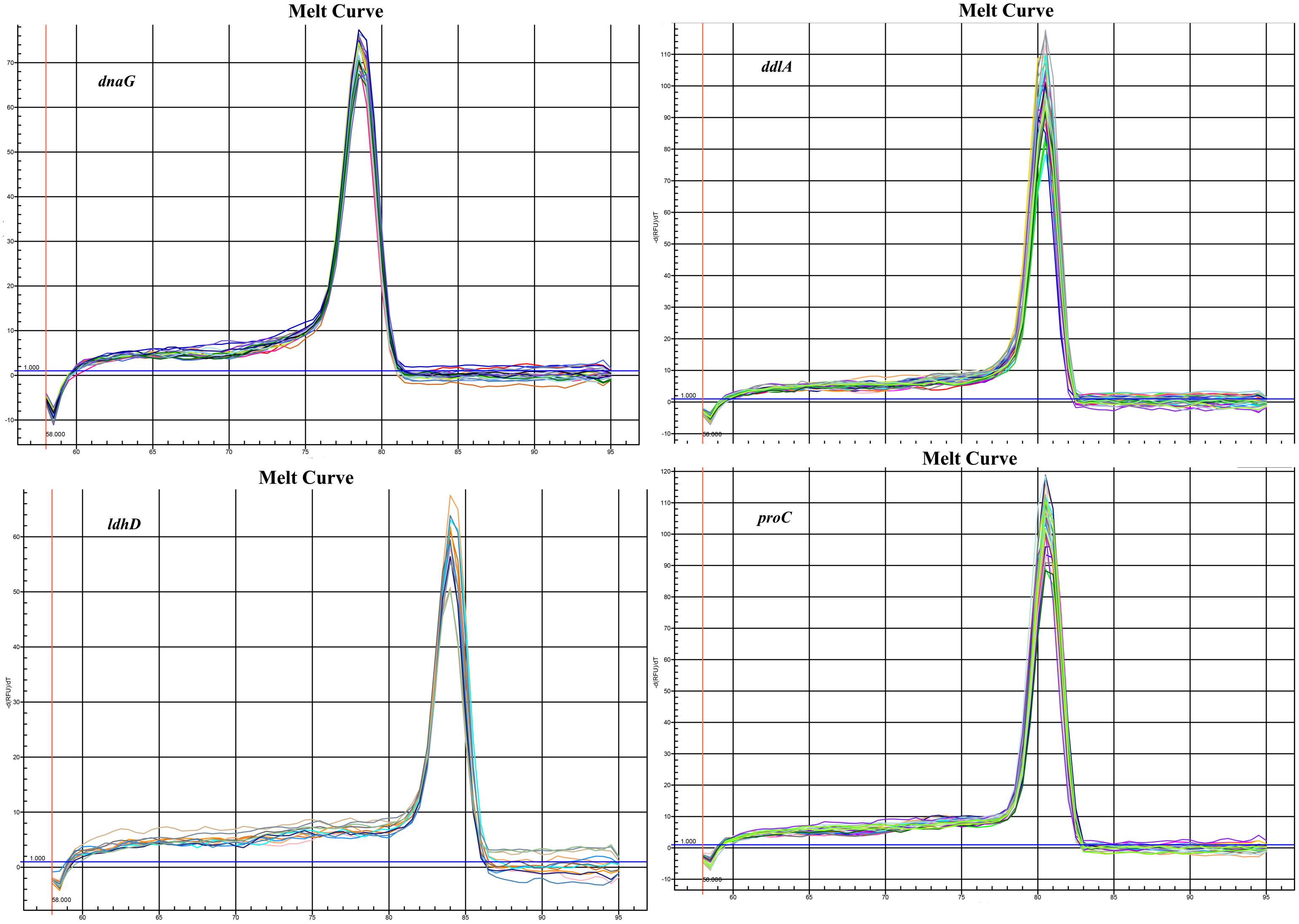

Melt Curve

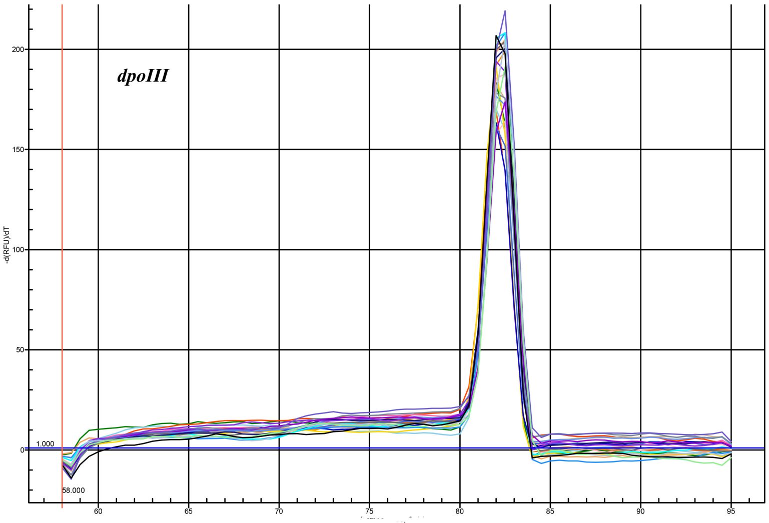

Melt Curve

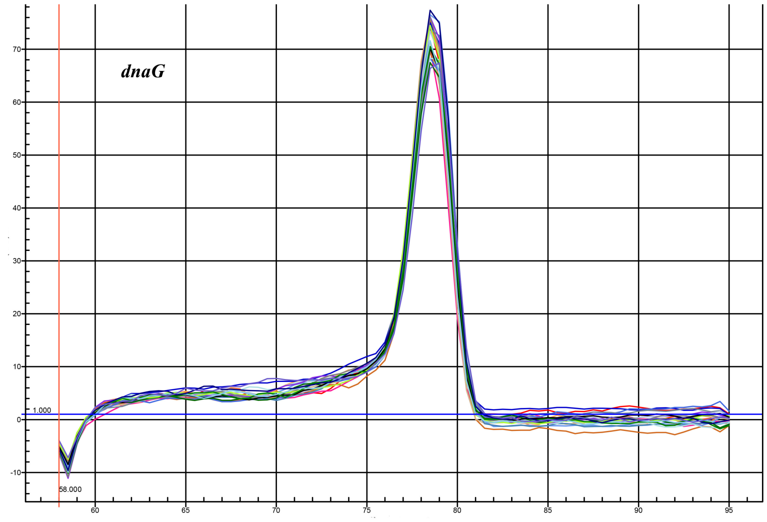

Melt Curve

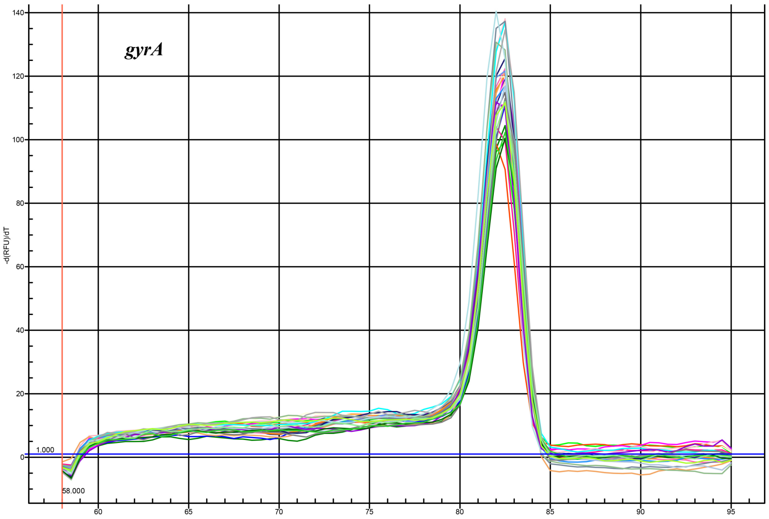

Melt Curve

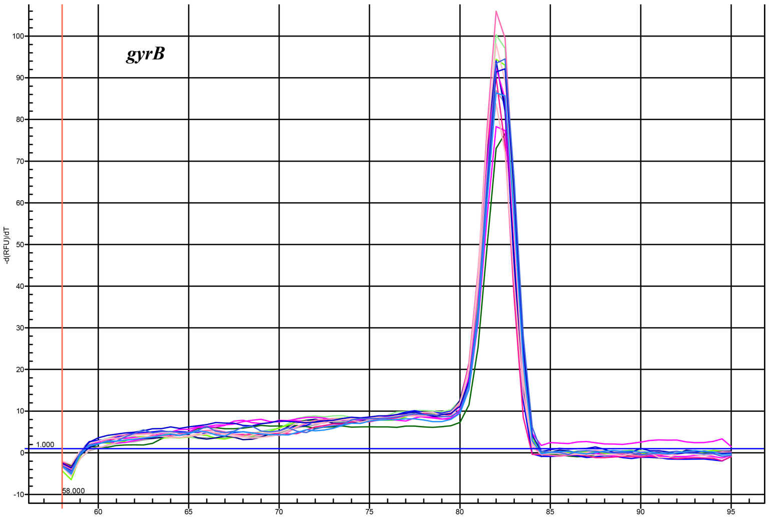

Melt curve

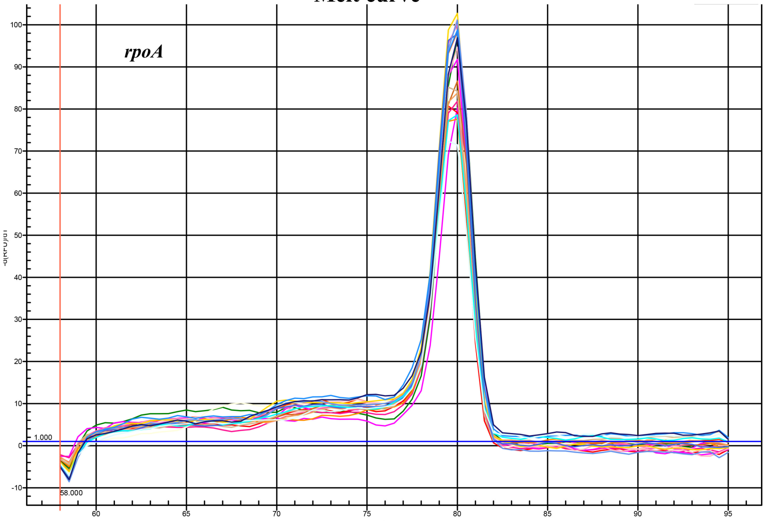

Melt Curve

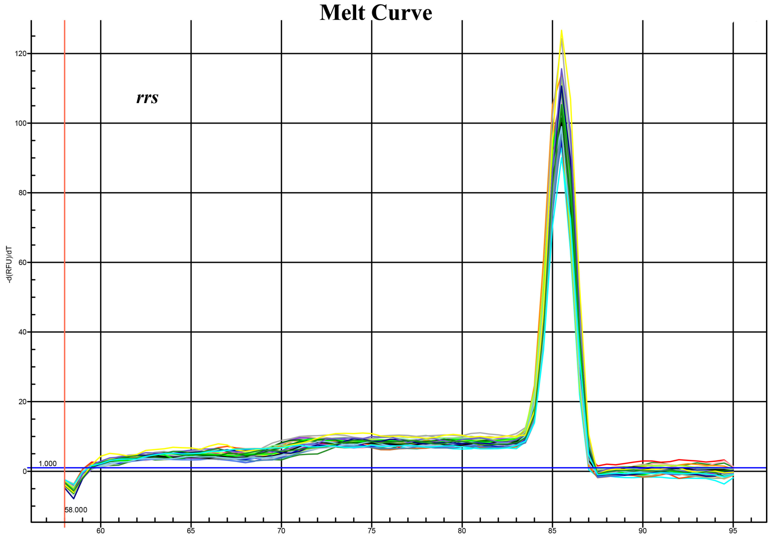

Supplement: Supplementary file 1 [file Image_1.PDF]
