# Supplementary material for: Selection and Validation of Reference Genes for Quantitative Real-Time PCR Normalization Under Ethanol Stress Conditions in Oenococcus oeni SD-2a
Source: Front Microbiol. 2018 May 4;9:892. doi: 10.3389/fmicb.2018.00892 (PMC5946679; doi:10.3389/fmicb.2018.00892)
Supplement: Supplementary file 2 [file Table_1.DOCX]

Supplementary Material

Selection and validation of reference genes for quantitative real-time PCR normalization under ethanol stress conditions in *Oenococcus oeni* SD-2a

**Shuai Peng, Longxiang Liu, Hongyu Zhao, Lin Yuan, Hua Wang^*^,** **Hua Li****^*^**

*** Correspondence:** Hua Li: lihuawine@nwafu.edu.cn Hua Wang: wanghua@nwafu.edu.cn

# Supplementary Table 1. Studies for evaluation of reference genes reported in *O. oeni*

| **Candidate reference gene** | **Stable Reference gene** | **Test condition** | **Methold** | **Reference** |
| --- | --- | --- | --- | --- |
| *ldhD, gyrA, gyrB, rpoD, dnaG, glk, rrs* | *ldhD* | Exponential phase, heat shock (42 ℃), ethanolic shock (11% vol/vol), acidic shock (pH 3.5), cold shock (18 ℃), synthetic wine, and stationary phase | 2^ΔCt^ | Desroche et al., 2005 |
|  |  |  |  |  |
| *ldhD, rpoB, dpoI, ddlA, gyrB, tkt, dpoIII, primG, pgm, purK* | *ddlA and gyrB* | Distilled water, MRS, wine-like medium(13% ethanol, 3.2 g/L L-malic acid and pH3.3) | geNorm , Normfinder and BestKeeper | Costantini et al., 2011 |
| *ldhD, gyrA, rpoA, gmk, recA, rpoB, ftsZ, pta, gyrB, gapA* | *ftsZ, pta, gapA, rpoA* | Ethanol (850 mM), butyric acid (25 mM), ethyl butyrate (10 mM), ethanol (850 mM) and butyric acid (25 mM) | geNorm | Sumby et al., 2012 |
|  |  |  |  |  |
| *rpoA, gyrB, gapA, gmk, recA, rpoB, ftsZ, pta, ldhD, rrs* | *pta, rpoB, gapA, recA, gyrB* | 7, 12, 13 and 15% ethanol | qbase+(geNorm) | Cafaro et al., 2014 |
| *ldhD,gyrA,rpoA,gmk, recA, rpoB, ftsZ, pta, gyrB, gapA* | *gmk, recA, gapA, ldhD* | 20% ethanol | qbase+(geNorm) | Betteridge et al., 2017 |
|  |  |  |  |  |
