# Supplementary material for: Selection and Validation of Reference Genes for Quantitative Real-Time PCR Normalization Under Ethanol Stress Conditions in Oenococcus oeni SD-2a
Source: Front Microbiol. 2018 May 4;9:892. doi: 10.3389/fmicb.2018.00892 (PMC5946679; doi:10.3389/fmicb.2018.00892)
Supplement: Supplementary file 3 [file Table_2.docx]

Supplementary Material

Selection and validation of reference genes for quantitative real-time PCR normalization under ethanol stress conditions in *Oenococcus oeni* SD-2a

**Shuai Peng, Longxiang Liu, Hongyu Zhao, Lin Yuan, Hua Wang,** **Hua Li^*^**

*** Correspondence:** Hua Li: lihuawine@nwafu.edu.cn Hua Wang: wanghua@nwafu.edu.cn

**Supplementary Table 2.** Ct values of nine candidate reference genes of *O.oeni* among 21 tested samples.

|  | *proC* | *rrs* | *dnaG* | *gyrA* | *ddlA* | *rpoA* | *gyrB* | *ldhD* | *dpoIII* |
| --- | --- | --- | --- | --- | --- | --- | --- | --- | --- |
| geo Mean [Ct] | 24.061 | 8.547 | 22.019 | 21.347 | 22.268 | 19.562 | 24.098 | 19.455 | 23.046 |
| ar Mean [Ct] | 24.088 | 8.568 | 22.032 | 21.364 | 22.281 | 19.604 | 24.120 | 19.498 | 23.061 |
| min [Ct] | 22.860 | 7.854 | 20.681 | 20.620 | 21.179 | 18.201 | 23.005 | 18.257 | 21.754 |
| max [Ct] | 26.099 | 9.470 | 23.733 | 23.057 | 23.048 | 21.644 | 25.949 | 21.700 | 24.749 |
| min [x-fold] | -2.299 | -1.617 | -2.527 | -1.656 | -2.127 | -2.569 | -2.132 | -2.295 | -2.449 |
| max [x-fold] | 4.107 | 1.896 | 3.283 | 3.271 | 1.717 | 4.235 | 3.607 | 4.740 | 3.256 |
| std dev [± x-fold] | 1.874 | 1.457 | 1.432 | 1.646 | 1.623 | 2.221 | 1.866 | 2.290 | 1.541 |
|  |  |  |  |  |  |  |  |  |  |
