# Supplementary material for: Selection and Validation of Reference Genes for Quantitative Real-Time PCR Normalization Under Ethanol Stress Conditions in Oenococcus oeni SD-2a
Source: Front Microbiol. 2018 May 4;9:892. doi: 10.3389/fmicb.2018.00892 (PMC5946679; doi:10.3389/fmicb.2018.00892)
Supplement: Supplementary file 4 [file Table_3.docx]

Supplementary Material

Selection and validation of reference genes for quantitative real-time PCR normalization under ethanol stress conditions in *Oenococcus oeni* SD-2a

**Shuai Peng, Longxiang Liu, Hongyu Zhao, Lin Yuan, Hua Wang^*^,** **Hua Li****^*^**

*** Correspondence:** Hua Li: lihuawine@nwafu.edu.cn Hua Wang: wanghua@nwafu.edu.cn

# Supplementary Table 3. GS values resulted from IdealRef

| Gene | GS Value |
| --- | --- |
| *gyrA/dpoIII* | 0.4939 |
| *dnaG* | 0.7442 |
| *ldhD* | 0.8622 |
| *gyrB* | 1.0692 |
| *proC* | 0.9710 |
| *rpoA* | 1.0360 |
| *ddlA* | 1.1256 |
| *rrs* | 1.1389 |
