# Supplementary material for: Selection and Validation of Reference Genes for Quantitative Real-Time PCR Normalization Under Ethanol Stress Conditions in Oenococcus oeni SD-2a
Source: Front Microbiol. 2018 May 4;9:892. doi: 10.3389/fmicb.2018.00892 (PMC5946679; doi:10.3389/fmicb.2018.00892)
Supplement: Supplementary file 5 [file Table_4.DOCX]

Supplementary Material

Selection and validation of reference genes for quantitative real-time PCR normalization under ethanol stress conditions in *Oenococcus oeni* SD-2a

**Shuai Peng, Longxiang Liu, Hongyu Zhao, Lin Yuan, Hua Wang^*^,** **Hua Li****^*^**

*** Correspondence:** Hua Li: lihuawine@nwafu.edu.cn Hua Wang: wanghua@nwafu.edu.cn

**Supplementary Table 4.** Parirwise variation (V value) resulted by geNorm

| V value | Total | 8% ethanol | 12% ethanol | 16% ethanol |
| --- | --- | --- | --- | --- |
| V2/3 | 0.175 | 0.099 | 0.187 | 0.117 |
| V3/4 | 0.204 | 0.223 | 0.137 | 0.123 |
| V4/5 | 0.17 | 0.143 | 0.158 | 0.125 |
| V5/6 | 0.133 | 0.143 | 0.122 | 0.109 |
| V6/7 | 0.122 | 0.122 | 0.126 | 0.103 |
| V7/8 | 0.108 | 0.122 | 0.153 | 0.123 |
| V8/9 | 0.131 | 0.171 | 0.137 | 0.15 |
