# Supplementary material for: Selection and Validation of Reference Genes for Quantitative Real-Time PCR Normalization Under Ethanol Stress Conditions in Oenococcus oeni SD-2a
Source: Front Microbiol. 2018 May 4;9:892. doi: 10.3389/fmicb.2018.00892 (PMC5946679; doi:10.3389/fmicb.2018.00892)
Supplement: Supplementary file 6 [file Table_5.DOCX]

Supplementary Material

Selection and validation of reference genes for quantitative real-time PCR normalization under ethanol stress conditions in *Oenococcus oeni* SD-2a

**Shuai Peng, Longxiang Liu, Hongyu Zhao, Lin Yuan, Hua Wang^*^,** **Hua Li^*^**

*** Correspondence:** Hua Li: lihuawine@nwafu.edu.cn Hua Wang: wanghua@nwafu.edu.cn

**Supplementary Table 5.** Relative expression levels of *hsp 18* in 8%, 12% and 16% ethanol ST calculated by 2^-ΔΔCt^methold. The sample groups(t=0h) were set as control groups. SEM: Standard Error of the Mean

| 8% ethanol | *dpoIII+gyrA* | SEM | *dpoIII and dnaG* | SEM | *ldhD* | SEM |
| --- | --- | --- | --- | --- | --- | --- |
| 0h | 1 | 0.435 | 1 | 0.4309 | 1 | 0.1078 |
| 1h | 14.183 | 5.03 | 11.916 | 4.279 | 44.5 | 18.192 |
| 3h | 168.098 | 19.186 | 133.614 | 27.927 | 404.516 | 164.469 |
| 12% ethanol | *gyrA+gyrB+rrs* | SEM | *dpoIII+gyrA+dnaG* | SEM | *rpoA* |  |
| 0h | 1 | 0.433 | 1 | 0.497 | 1 | 0.497 |
| 1h | 4.915 | 0.429 | 2.247 | 0.368 | 7.401 | 1.248 |
| 3h | 13.562 | 2.009 | 9.54 | 1.548 | 67.536 | 12.307 |
| 16% ethanol | *proC+gyrB* | SEM | *dnaG+dpoIII* | SEM | *ldhD* | SEM |
| 0h | 1 | 0.436 | 1 | 0.431 | 1 | 0.436 |
| 1h | 139.507 | 13.375 | 43.595 | 7.867 | 70.467 | 11.883 |
| 3h | 132.43 | 14.623 | 119.342 | 15.968 | 231.375 | 36.049 |
